# Modelling substrate specificity and enantioselectivity for lipases and esterases by substrate-imprinted docking

**DOI:** 10.1186/1472-6807-9-39

**Published:** 2009-06-03

**Authors:** P Benjamin Juhl, Peter Trodler, Sadhna Tyagi, Jürgen Pleiss

**Affiliations:** 1 Institute of Technical Biochemistry, University of Stuttgart, Allmandring 31, 70569 Stuttgart, Germany

## Abstract

**Background:**

Previously, ways to adapt docking programs that were developed for modelling inhibitor-receptor interaction have been explored. Two main issues were discussed. First, when trying to model catalysis a reaction intermediate of the substrate is expected to provide more valid information than the ground state of the substrate. Second, the incorporation of protein flexibility is essential for reliable predictions.

**Results:**

Here we present a predictive and robust method to model substrate specificity and enantioselectivity of lipases and esterases that uses reaction intermediates and incorporates protein flexibility. Substrate-imprinted docking starts with covalent docking of reaction intermediates, followed by geometry optimisation of the resulting enzyme-substrate complex. After a second round of docking the same substrate into the geometry-optimised structures, productive poses are identified by geometric filter criteria and ranked by their docking scores. Substrate-imprinted docking was applied in order to model (i) enantioselectivity of ***Candida antarctica ***lipase B and a W104A mutant, (ii) enantioselectivity and substrate specificity of ***Candida rugosa ***lipase and ***Burkholderia cepacia ***lipase, and (iii) substrate specificity of an acetyl- and a butyrylcholine esterase toward the substrates acetyl- and butyrylcholine.

**Conclusion:**

The experimentally observed differences in selectivity and specificity of the enzymes were reproduced with an accuracy of 81%. The method was robust toward small differences in initial structures (different crystallisation conditions or a co-crystallised ligand), although large displacements of catalytic residues often resulted in substrate poses that did not pass the geometric filter criteria.

## Background

The number of protein structures available to researchers has grown exponentially over the last two decades and more than 50 000 experimentally determined structure entries are now held in the Protein Data Bank [1]. Furthermore, comparative structure prediction allows to derive reliable structure models from sequence information [2]. In silico methods are being developed to predict affinity, activity, specificity, and selectivity of newly discovered proteins based on structure information [3]. In drug development, molecular docking is routinely used to identify new lead compounds by virtual screening of libraries of small compounds [4]. Recently, docking methods have also been successfully applied to predict the most probable substrates of enzymes with unknown function, but known structure [5,6]. Previously, the specificity of enzymes was investigated by non-covalent docking of putative metabolites into the substrate binding site [7] and substrates for short chain dehydrogenases/reductases were identified by molecular docking [8]. A similar method was used to identify eight new substrates for *Pseudomonas diminuta *phosphotriesterase [9]. Use of an improved scoring function made it possible to predict relative binding free energies for *α*-*β *barrel proteins and their metabolites [10]. The docking results were further improved for protein structures which had been resolved without a ligand by a restricted energy minimisation of the binding pocket around the docked metabolite. While all these methods considered the ground state of the substrate, reaction intermediates of putative substrates were successfully used to predict substrates of amidohydrolases [11], and docking of transition-states of flunitrazepam and progesterone have been docked into cytochrome P450 monooxygenases to predict hydroxylation patterns [12]. Especially these two later findings support our approach of focusing on reaction intermediates when docking substrates into enzymes. Carboxylic ester hydrolases (EC 3.1.1) are a large family of industrially relevant biocatalysts because they have been shown to catalyse hydrolysis of ester substrates with high regio- and enantioselectivity as well as the reverse reaction, the acylation of alcohols [13-15]. Their reaction mechanism is well understood [16,17]: Upon nucleophilic attack of the catalytic serine, a tetrahedral intermediate is formed which is considered the rate limiting step. The binding pockets of esterases provide a pre-organised environment to specifically stabilise this intermediate by hydrogen-bonding. Therefore, a predictive model for esterase substrates has to take into account the following points:

1. The substrate has to be covalently docked to the enzyme in its tetrahedral intermediate state. While docking of molecules in their ground state allows predictions of the binding of that molecule to an enzyme, it does not allow to draw direct conclusions whether the molecule is converted by the enzyme or not. A docking method that aims to model enzymatic catalysis should reflect the molecular role of the enzyme in stabilising the transition-state [18]. A tetrahedral intermediate that is covalently bound to the catalytic serine is very close to the transition state which is formed during the enzyme-catalysed ester hydrolysis [19]. Since in both states the interactions of the enzyme with the acid moiety as well as with the alcohol moiety are identical, the tetrahedral intermediate is considered to be appropriate to predict the relative catalytic activity towards different substrates.

2. In addition, the docking pose of a putative substrate is essential. In order to be converted, the hydrogen bond network stabilising the intermediate has to be fully formed. Therefore, a simple geometric filter allows to distinguish between productive and non-productive substrate poses [20,21].

3. X-ray structures and structure models based on homology are often not in a conformation to accommodate putative substrates, because even small differences in structures can have a strong effect on molecular docking results [22]. To overcome this problem, it is necessary to introduce protein flexibility into the docking procedure, allowing the enzyme to adjust its conformation to the substrate. Current docking programs treat the ligand as a flexible molecule, but consider the protein to be rigid. Ways to account for protein flexibility are a point of focus in current molecular docking research and a variety of methods have been suggested [23]. Methods that incorporate limited flexibility for the proteins allow the receptor to bend in hinge regions [22], introduce a limited flexibility of amino acid side chains in the active site [24,25], or change the allowed overlap between ligand and protein [26]. Other docking methods represent protein flexibility by different protein structures or a rotamer library of substrate-interacting residues. The ligand is docked either into an ensemble of protein structures [27,28], into an averaged structure [29], or into a pharmacophore grid [30]. However, this limited flexibility is not able to account for all possible conformational changes that occur in proteins upon ligand binding [31]. A fully flexible protein can be simulated by molecular mechanics/molecular dynamics and Monte Carlo methods. Molecular dynamics simulations of a defined binding site [12] or the whole ligand-protein complex [32] have been applied to improve docking results from rigid protein docking. Similarly, all-atom Monte Carlo docking algorithms have been successfully used to model drug-DNA binding [33].

Here we introduce a strategy of substrate-imprinted docking, which uses the docking program FlexX [34,35], geometric filter criteria, and structure optimisation by molecular mechanics to account for full protein flexibility. The capability of this strategy was assessed in a case study on several lipases and two esterases to model enantioselectivity and substrate specificity:

• The wild type of *Candida antarctica *lipase B (CALB) was compared to a mutant (W104A) with altered enantioselectivity [36] by docking the two enantiomers of 1-phenylethyl butyrate ((*R*)-PEB and (*S*)-PEB) to model enantioselectivity.

• The enantiomers of 2- to 8-methyldecanoic acid butyl esters ((*R/S*)-2- to (*R/S*)-8-MDB) were docked into *Candida rugosa *lipase (CRL) to assess the capabilities of modelling lower enantioselectivities.

• CRL and *Burkholderia cepacia *lipase (BCL) were compared by docking the enantiomers of 2-hydroxyoctanoic acid butyl ester ((*R/S*)-2-HOB) and 2- to 4-methylpentanoic acid pentyl esters ((*R/S*)-2-MPP, (*R/S*)-3-MPP, 4-MPP) in order to model enantioselectivity and substrate specificity.

• *Torpedo californica *acetylcholine esterase (TcAChE) was compared to the human butyrylcholine esterase (huBuChE) by docking of acetylcholine (ACh) and butyrylcholine (BuCh) to model substrate specificity.

## Results

### Docking esters of chiral secondary alcohols into C. antarctica lipase B structures

#### Conventional docking

Tetrahedral reaction intermediates were covalently docked into CALB and its W104A mutant. During docking, the protein structure was assumed to be rigid, while the docked substrate was treated flexible. The docking procedure consists of three steps: (i) the construction of the putative substrates in their tetrahedral intermediate forms, (ii) the covalent docking into the active site, and (iii) the application of the geometric filter criteria for docked substrate poses. (*R*)-PEB and (*S*)-PEB were docked into five X-ray structures of CALB and the five models of its W104A mutant. Experimentally, CALB shows a enantiopreference in transesterification toward the (*R*)-enantiomer of PEB with a very high E-value of 1 300 000 [36], while the W104A mutant is non-selective. While all the structures were highly similar (all-atom RMSDs between the structures were less than 0.5 Å), the docking scores differed considerably (Table 1 ans see Additional file 1, S11). For four structures productive poses for a reaction intermediate of (*R*)-PEB were found. For the structure 1TCB no productive pose could be found by docking, which corresponds to a false negative result. For four structures no productive pose was found for the reaction intermediate of (*S*)-PEB, while a productive pose was found for 1LBT (false positive). Thus, the accuracy for the wild type without optimising the geometry is 80% – eight correct predictions, one false negative and one false positive.

**Table 1:** Docking of 1-phenylethyl butyrate

	Docking into:			
	X-ray structures		Substrate-imprinted structures	
		
Structure	(*R*)-PEB	(*S*)-PEB	(*R*)-PEB	(*S*)-PEB
*Candida antarctica *lipase B				
Experimental data	+	-	+	-
1LBS^*a*^	+	-	+	+
1LBT^*a*^	+	+	+	-
1TCA	+	-	+	-
1TCB	-	-	+	-
1TCC	+	-	+	-
No. false predictions	1	1	0	1

*Candida antarctica *lipase B, W104A mutant				
Experimental data	+	+	+	+
1LBSW104A^*a*^	+	-	+	+
1LBTW104A^*a*^	-	-	+	+
1TCAW104A	+	-	+	+
1TCBW104A	+	-	+	+
1TCCW104A	+	-	+	+
No. false predictions	1	5	0	0

Docking of (*R*)-PEB and (*S*)-PEB into five X-ray structures of CALB and five structure models of a W104A mutant of CALB using FlexX. The substrates were docked into the non-optimised X-ray structures and the substrate-imprinted structures. "+" and "-" indicate that the docking results predict (*R*)-PEB or (*S*)-PEB to be a substrate or a non-substrate. Correct predictions are indicated by bold and large font type. Experimental data [36] is included for comparison. ^*a *^Structure was resolved with an inhibitor bound.

The same docking procedure was performed with the five models of the W104A mutant. In four models (*R*)-PEB could be docked in a productive pose (Table 1), while no productive pose could be found for 1LBTW104A (false negative). For the (*S*)-enantiomer of PEB no productive pose could be found for any of the five mutant structures. This corresponds to five false negative results, because experimentally the (*S*)-enantiomer of PEB is converted as efficiently as the (*R*)-enantiomer. Thus, the accuracy for the mutant without optimising the geometry is 40% – four correct predictions and six false negatives.

In previous studies [37], protein structures that were resolved with a particular ligand tended to give good docking results for similar ligands or ligands that have a similar mode of binding, while protein structures without inhibitor or in complex with a structurally different inhibitor failed more often. For docking of PEB into CALB and its mutant, structures with and without inhibitor have similar predictive accuracies. As expected, structures without a bound inhibitor have a tendency to lead to false negatives, such as for docking of (*R*)-PEB into 1TCB, while structures with inhibitor have a tendency to lead to false positives, such as docking of (*S*)-PEB into 1LBT. This is caused by small differences in the structures, which lead to large differences in docking scores, as previously observed for trypsin, thrombin, and HIV-1-protease [38]. To overcome these limitations of protein rigidity [39] and to increase the accuracy, the docking procedure has to take into account protein flexibility.

#### Substrate-imprinted docking

To account for protein flexibility, protein-substrate complexes obtained by docking were subsequently optimised by energy minimisation. The resulting geometry-optimised structures of the protein are referred to as substrate-imprinted structures and were then used in a second round of covalent docking of the same substrate. The resulting poses were then analysed for the geometric filter criteria, the docking score, and the overlap volume (Table 1). Docking of (*R*)-PEB into CALB wild type resulted in productive poses for all five CALB structures. In contrast, docking of (*S*)-PEB led only for one structure (1LBS) to a productive pose (false positive). Thus, the accuracy of substrate-imprinted docking increased to 90% (nine correct predictions and one false positive) as compared to 80% for conventional docking, and the deviation between the docking scores was slightly reduced from 2.0 kJ/mol to 1.7 kJ/mol [see Additional file 1, S11]. In contrast to docking into the X-ray structures, no false negative result was found. While docking of (*R*)-PEB into the X-ray structure 1TCB led to a false negative result, substrate-imprinted docking based on 1TCB led to a productive pose. Similarly, the productive pose upon docking of (*S*)-PEB into the X-ray structure 1LBT (false positive result) was not found upon substrate-imprinted docking, but a new false positive result was found (1LBS).

The largest impact of substrate-imprinted docking was observed for the mutant W104A. Here, docking into rigid model structures failed in six out of ten cases. However, docking of (*R*)-PEB into substrate-imprinted mutant structures resulted in productive poses for all five structures. Similarly, substrate-imprinted docking of (*S*)-PEB also led to productive poses for all structures. This result for the mutant is in agreement with experimental observations and corresponds to an accuracy of 100% – ten correct predictions.

**Figure 1:**
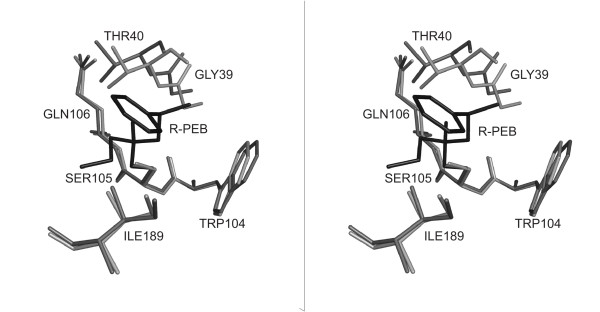
**Binding pocket of C. antarctica lipase B with docked substrate (*R*)-PEB**. Stereo image of the alcohol binding pocket of 1TCB (light grey), the alcohol pocket of the substrate-imprinted model of 1TCB (dark grey) and the highest scored productive pose of (*R*)-PEB (black), docked into the substrate-imprinted model.

The structural changes upon geometry optimisation are generally small. This also applies to the optimisation of the structure 1TCB (Fig. 1), which led to a false negative result upon docking of (*R*)-PEB into the X-ray structure, while substrate-imprinted docking found a productive pose. However, these small conformational changes in the alcohol binding pocket (all-atom RMSD of 0.46 Å, Table 2) are sufficient to remove clashes between the docked substrate and the enzyme, especially in complexes where the substrate moieties fit tightly into buried protein pockets, and thus allow to dock (*R*)-PEB in a productive pose. These changes in the alcohol binding pocket are in the same range as the overall conformational changes upon geometry optimisation (between 0.36 Å and 0.45 Å) for the CALB structures (Table 2). Previously it has been shown that a side chain optimisation was sufficient to successfully dock inhibitors into kinase structures [40]. This method needs a X-ray structure of the inhibitor under investigation with a homologous protein as a starting point and assumes a rigid backbone. In contrast, substrate-imprinted docking can be applied to docking of new substrates and is able to improve binding pockets which are partially formed by backbone atoms, such as the oxyanion hole of lipases and esterases. For a typical substrate-enzyme complex, such a full geometry optimisation takes less than 15 minutes on a dual core 2.0 GHz Opteron CPU.

**Table 2:** RMSD of C. antarctica lipase B alcohol pocket after Optimization

	All-atom RMSD [Å]	
	total	alcohol pocket
	
1LBS/(*R*)-PEB	0.45	0.41 (92%)
1LBS/(*S*)-PEB	0.45	0.68 (150%)^*a*^
1LBT/(*R*)-PEB	0.39	0.41 (105%)
1LBT/(*S*)-PEB	0.41	0.69 (170%)
1TCA/(*R*)-PEB	0.36	0.40 (110%)
1TCA/(*S*)-PEB	0.36	0.37 (103%)
1TCB/(*R*)-PEB	0.36	0.46 (121%)
1TCB/(*S*)-PEB	0.36	0.43 (118%)
1TCC/(*R*)-PEB	0.40	0.44 (109%)
1TCC/(*S*)-PEB	0.39	0.41 (105%)
1LBSW104A/(*R*)-PEB	0.35	0.52 (149%)
1LBSW104A/(*S*)-PEB	0.33	0.21 (65%)
1LBTW104A/(*R*)-PEB	0.33	0.29 (87%)
1LBTW104A/(*S*)-PEB	0.34	0.28 (83%)
1TCAW104A/(*R*)-PEB	0.28	0.28 (100%)
1TCAW104A/(*S*)-PEB	0.31	0.25 (80%)
1TCBW104A/(*R*)-PEB	0.3	0.33 (109%)
1TCBW104A/(*S*)-PEB	0.32	0.23 (72%)
1TCCW104A/(*R*)-PEB	0.31	0.33 (105%)
1TCCW104A/(*S*)-PEB	0.29	0.24 (84%)

All-atom RMSD of the alcohol pocket of CALB in comparison to the all-atoms RMSD of the whole protein after geometry optimisation. The percentage values in brackets represent the alcohol pocket RMSD as a percentage of the all-atoms RMSD of the whole protein. ^*a *^False prediction.

### Docking esters of chiral and non-chiral carboxylic acids into CRL and BCL structures

#### Conventional docking

Tetrahedral reaction intermediates of 2- to 8-MDB were docked into seven CRL X-ray structures (two of those structures had a displaced histidine) in order to model enantiopreference. Similar intermediates of 2-HOB and 2- to 4-MPP were docked into the same CRL structures and seven BCL X-ray structures in order to model substrate specificity. It has been shown experimentally that 2- to 8-MDBs can be synthesised by CRL with E-values between 2.8 an 91, alternately preferring the (*R*)- or the (*S*)-enantiomer [41]. 2-HOB is synthesised by CRL and BCL, with a preference for the (*R*)-enantiomer (E-values of about 20) [42], 4-MPP is synthesised by CRL and BCL, 3-MPP is synthesised by neither CRL nor BCL, and 2-MPP is synthesised by CRL, but not BCL [43].

Docking both enantiomers of 2- to 8-MDBs into CRL did most often result in predictions, that were either positive or negative for both enantiomers (Table 3). Thus, no stereoselectivity could be seen in the docking results. In particular, docking into the two structures 1LPN and 1LPP never resulted in a productive pose, due to the displacement of the catalytic histidine in these structures. For (*R*)-2-MDB productive poses could only be found for two structures, while for the (*S*)-enantiomer, a productive pose could only be found for one structure. Thus, productive poses for MDBs were only found in 42% of the cases (41 correct predictions, 57 false negatives) and no enantiopreference could be observed in the docking results. The E-values CRL and 2- to 8-MDB are much lower than those observed in the case of CALB and PEB (E-value = 1 300 000), and the synthesis of the less prefered enantiomer did still occur. Therefore, both enantiomers were considered to be experimentally validated substrates for CRL and BCL.

**Table 3:** Docking of methyldecanoic acid butyl esters

	Experimental data	Docking into X-ray structures							No. of false predictions
Substrate		1CLE^*a*^	1CRL	1LPM^*a*^	1LPN^*a*, *b*^	1LPO^*a*^	1LPP^*a*, *b*^	1LPS^*a*^	
(*R*)-2-MDB	+	+	-	-	-	+	-	-	5
(*S*)-2-MDB	+	+	-	-	-	-	-	-	6
(*R*)-3-MDB	+	+	+	-	-	+	-	-	4
(*S*)-3-MDB	+	+	+	-	-	+	-	-	4
(*R*)-4-MDB	+	+	+	-	-	+	-	-	4
(*S*)-4-MDB	+	+	+	-	-	+	-	-	4
(*R*)-5-MDB	+	+	+	-	-	+	-	-	4
(*S*)-5-MDB	+	+	+	-	-	+	-	-	4
(*R*)-6-MDB	+	+	+	+	-	+	-	-	3
(*S*)-6-MDB	+	+	+	+	-	+	-	-	3
(*R*)-7-MDB	+	+	+	-	-	+	-	-	4
(*S*)-7-MDB	+	+	+	-	-	+	-	-	4
(*R*)-8-MDB	+	+	+	-	-	+	-	-	4
(*S*)-8-MDB	+	+	+	-	-	+	-	-	4

Docking into substrate-imprinted structures									
(*R*)-2-MDB	+	+	+	+	-	+	-	-	3
(*S*)-2-MDB	+	-	+	+	-	+	-	-	4
(*R*)-3-MDB	+	+	+	+	-	+	-	-	3
(*S*)-3-MDB	+	-	+	+	-	+	-	-	4
(*R*)-4-MDB	+	+	+	+	-	+	-	-	3
(*S*)-4-MDB	+	+	+	+	-	+	-	+	2
(*R*)-5-MDB	+	+	+	+	-	+	-	+	2
(*S*)-5-MDB	+	+	+	+	-	+	-	+	2
(*R*)-6-MDB	+	+	+	+	-	+	-	+	2
(*S*)-6-MDB	+	+	+	+	-	+	-	-	3
(*R*)-7-MDB	+	+	+	+	-	+	-	-	3
(*S*)-7-MDB	+	+	+	+	-	+	-	-	3
(*R*)-8-MDB	+	+	+	+	-	+	-	-	3
(*S*)-8-MDB	+	+	+	+	-	+	-	-	3

Docking of 2- to 8-MDB into seven CRL structures using FlexX. The substrates were docked into the not optimised X-ray structures and the substrate-imprinted structures. "+" and "-" indicate that the docking results predict 2- to 8-MDB to be a substrate or a non-substrate. Correct predictions are indicated by bold and large font type. Experimental data [41] is included for comparison. ^*a *^Structure was resolved with an inhibitor bound. ^*b *^Displaced histidine.

Docking 2-HOB into CRL and BCL resulted in productive poses in most cases, but no distinction between the two enantiomers could be made. The experimentally observed E-value [42] was in the range of the E-values observed for CRL and 2- to 8- MDB, and both enantiomers were therefore considered to be experimentally converted substrates, too. For four CRL structures productive poses for the (*R*)-enantiomer and the (*S*)-enantiomer could be found (Table 4). No productive poses for any enantiomer could be found when docking into the other three CRL structures (1LPN, 1LPP, 1LPS). Productive poses for both enantiomers were also found for five BCL structures, while for two structures no productive poses could be found. 2-HOB was correctly identified as a substrate with an accuracy of 64% – 18 correct predictions, and 10 false negatives, but no enantiopreference could be observed in the docking results.

**Table 4:** Docking of 2-hydroxyoctanoic acid butyl esters

	Docking into:			
	X-ray structures		Substrate-imprinted structures	
		
Structure	(*R*)-2-HOB	(*S*)-2-HOB	(*R*)-2-HOB	(*S*)-2-HOB
*Candida rugosa *lipase				
Experimental data	+	+	+	+
1CLE^*a*^	+	+	+	+
1CRL	+	+	+	+
1LPM^*a*^	+	+	+	+
1LPN^*a*, *b*^	-	-	-	-
1LPO^*a*^	+	+	+	+
1LPP^*a*, *b*^	-	-	-	-
1LPS^*a*^	-	-	-	+
No. false predictions	3	3	3	2

*Burkholderia cepacia *lipase				
Experimental data	+	+	+	+
2LIP	-	-	-	+
3LIP	-	-	+	-
4LIP^*a*^	+	+	+	+
5LIP^*a*^	+	+	+	+
1OIL	+	+	+	+
1YS1^*a*^	+	+	+	+
1YS2^*a*^	+	+	+	+
No. false predictions	2	2	1	1

Docking of (*R*)-2-HOB and (*S*)-2-HOB into seven BCL and seven CRL structures using FlexX. The substrates were docked into the not optimised X-ray structures and the substrate-imprinted structures. "+" and "-" indicate that the docking results predict (*R*)-2-HOB or (*S*)-2-HOB to be a substrate or a non-substrate. Correct predictions are indicated by bold and large font type. Experimental data [42] is included for comparison. ^*a *^Structure was resolved with an inhibitor bound. ^*b *^Displaced histidine.

Docking 2- to 4-MPP into CRL X-ray structures resulted in only 17 correct predictions [see Additional file 2], where neither the substrates 2-MPP and 4-MPP nor the non-substrate 3-MPP were correctly predicted. When docking into the seven BCL X-ray structures, the substrate 4-MPP resulted in productive poses, and the non-substrates 2-MPP and 3-MPP also resulted in productive poses in many cases, leading to 21 false predictions. For eight structures productive poses were always found, no matter whether a substrate or a non-substrate was docked. For four structures (1LPN, 1LPP, 1LPS, 2LIP) no productive pose was found, regardless of the docked ligand. Docking into crystal structures of CRL and BCL is therefore not able to differentiate between substrates and non-substrates in the case of MPPs. Thus the experimentally described substrates 4-MPP for CRL and BCL and 2-MPP for CRL were correctly modelled with an accuracy of 67%, while the non-substrates 3-MPP for CRL and BCL and 2-MPP for BCL were correctly modelled with an accuracy of 33%. The overall accuracy for docking MPP was 44% – 31 correct predictions, 11 false negatives, and 28 false positives,

#### Substrate-imprinted docking

The capabilities of molecular docking to identify substrates and non-substrates were improved by using the method of substrate-imprinted docking. Docking 2- to 8-MDBs into substrate-imprinted CRL structures led to 58 productive poses (Table 3). The two structures with the displaced histidine (1LPN, 1LPP) did not provide any productive poses, as was already observed for the conventional docking. Thus, the identification of these esters as substrates was improved by substrate-imprinted docking to an accuracy of 59%, compared to the accuracy of 42% that was achieved with conventional docking. In contrast, substrate-imprinted docking was not able to identify enantioselectivities in the case of CRL and MDBs. When 2-HOB was docked into substrate-imprinted CRL structures, four productive poses could be found for the (*R*)-enantiomer and five for the (*S*)-enantiomer (Table 4). When using substrate-imprinted BCL structures, six productive poses were found for (*R*)-2-HOB and six productive poses were found for the (*S*)-enantiomer. Thus, substrate-imprinted docking improved the identification of 2-HOB as a substrate for CRL and BCL from 64% to 75%, but did not result predictions that reflected the experimentally determined enantioselectivity (E ≈ 20).

Docking 2-MPP into substrate-imprinted CRL structures resulted in two productive poses for the (*S*)-enantiomer and none for the (*R*)-enantiomer [see Additional file 2]. When docking into substrate-imprinted BCL structures, four productive poses were found for the (*R*)-enantiomer, and none for the (*S*)-enantiomer. No productive poses could be found for docking 3-MPP into substrate-imprinted CRL structures, three productive poses could be found for each enantiomer when docking 3-MPP into substrate-imprinted BCL structures. When docking 4-MPP into substrate-imprinted CRL structures, five productive poses were found. For the structures 1LPN and 1LPP, no productive poses were found. When docking 4-MPP into substrate-imprinted BCL structures, productive poses were found for all seven structures. Substrate-imprinted docking was therefore able to identify the substrates 4-MPP for CRL and BCL, and 2-MPP for CRL with an accuracy of 50%. However, while the recognition of 4-MPP as a substrate was improved by substrate-imprinted docking, the recognition of 2-MPP as a substrate was better by conventional docking. The non-substrates 3-MPP for CRL and BCL and 2-MPP for BCL were correctly modelled with an accurracy of 76%. Thus, substrate-imprinted docking can, in the case of MPPs and CRL/BCL, differentiate between substrates and non-substrates with an accurracy of 66%, while conventional docking only achieved an accurracy of 44%.

When docking into the CRL structure 1LPP and its substrate-imprinted forms, no productive pose could be found for any of the docked molecules, and when using the structure 1LPN, the only productive pose found was for 2-HOB. A closer examination of these two X-ray structures reveals that in both of them a inhibitor is bound to the catalytic serine, and a second inhibitor molecule is bound to the catalytic histidine [44]. Because of this, the catalytic histidine (H449) in both structures is displaced by 3.1 Å when compared to the X-ray structure 1CRL. Such a large displacement was not corrected during the geometry optimisation.

### Docking acetylcholine and butyrylcholine into AChE and BuChE structures

#### Conventional docking

In order to evaluate the capabilities of this method to correctly model substrate specificity with X-ray structures, tetrahedral reaction intermediates of ACh and BuCh were covalently docked into six TcAChE X-ray structures and four huBuChE X-ray structures. TcAChE only converts esters with a small acetyl moiety, because the acyl pocket of the protein is small. Therefore, TcAChE activity toward butyrylthiocholine is 850-fold lower than toward acetylthiocholine [45]. In contrast, huBuChE has a similar activity towards ACh and BuCh, because of its larger acyl pocket [46,47].

Conventional docking into TcAChE and huBuChE did not differentiate between the two substrates. No docking solution could be found with two TcAChE structures and two huBuChE structures, while all other structures provided productive poses for both substrates (Table 5). The accuracy of conventional docking was 50% – 10 correct predictions, six false negatives, and four false positives.

**Table 5:** Docking of acetylcholine and butyrylcholine

	Docking into:			
	X-ray structures		Substrate-imprinted structures	
		
Structure	ACh	BuCh	ACh	BuCh
*Torpedo californica *acetylcholine esterase				
Experimental data	+	-	+	-
1CFJ^*a*^	+	+	+	-
1DX6^*a*^	+	+	+	+
1E3Q^*a*^	-	-	+	-
1EVE^*a*^	+	+	+	-
1VXR^*a*, *b*^	-	-	-	-
1QIM	+	+	+	-
No. false predictions	2	4	1	1

human butyrylcholine esterase				
Experimental data	+	+	+	+
1P0M	-	-	-	-
1XLU^*a*^	+	+	+	+
1XLV^*a*^	+	+	+	+
1XLW^*a*^	-	-	+	+
No. false predictions	2	2	1	1

Docking of ACh and BuCh into six X-ray structures of the acetylcholine esterase from *Torpedo californica *and four X-ray structures of the human butyrylcholine esterase using FlexX. The substrates were docked into the not optimised X-ray structures and the substrate-imprinted structures. "+" and "-" indicate that the docking results predict ACh or BuCh to be a substrate or a non-substrate. Correct predictions are indicated by bold and large font type. Experimental data [45,46] is included for comparison. ^*a *^Structure was resolved with an inhibitor bound. ^*b *^Displaced histidine.

While the docking results differ considerably, the differences between the structures of each enzyme are small. The RMSD of the backbone atoms between the six TcAChE or between four huBuChE X-ray structures is below 0.5 Å and 0.4 Å respectively. Co-crystallised inhibitors had no influence on the ability to find productive substrate poses. While the two TcAChE structures that did not lead to a productive pose (1E3Q, 1VXR) had been resolved with inhibitors, the TcAChE structure that had been resolved without inhibitor led to productive poses. Similarly, the huBuChE structure that had been resolved in complex with a choline ligand (1P0M) did not lead to productive poses, as well as one of the structures that was resolved with an inhibitor.

#### Substrate-imprinted docking

To improve predictability of substrate specificity, substrate-imprinted docking was applied. Docking ACh into substrate-imprinted TcAChE structures led to five productive poses (Table 5). It was not possible to dock ACh into the substrate-imprinted structure 1VXR (false negative). When docking BuCh into substrate-imprinted TcAChE structures, five of the six structures did not bind BuCh in a productive pose, while the substrate-imprinted structure 1DX6 led to a productive pose for BuCh (false positive).

Substrate-imprinted huBuChE structures led to productive poses for ACh and BuCh in three out of four cases. The substrate-imprinted structure 1P0M did not lead to a productive pose for any of the substrates. Thus, substrate-imprinted docking into TcAChE and huBuChE achieved an overall accuracy of 80% (16 correct predictions, three false negatives, and one false positive), while docking into structures that had not been optimised to fit the docked substrates only achieved an accuracy of 50%. In addition to the higher accuracy, substrate-imprinted docking resulted in lower docking scores and a smaller spread of docking scores of true positive results [see Additional file 1, S12].

## Discussion

### Accuracy of the method

It has been shown that substrate specificity and enantioselectivity of lipases and esterases are a consequence of a delicate balance between enthalpic and entropic contributions [48]. While shape fitting and enthalpic terms are well represented by substrate-imprinted docking, entropic contributions are only partially accounted for in the scoring function of FlexX. Previously, improved scoring functions have been proposed [10]. In addition, it has been observed for lipases that different organic solvents can mediate the experimentally determined enantioselectivity [49,50]. However, none of the docking methods used today accounts for the molecular effects of organic solvents.

Beside the energy minimisation used in substrate-imprinted docking in order to optimise the structure of the substrate-enzyme complex, there are other more computational intensive methods like molecular dynamics or simulated annealing available that could be employed for the optimisation. However, clashes between atoms can easily be relaxed by a simple energy minimisation. In fact, such a minimisation is performed in many molecular dynamic protocols prior to the simulation itself for the purpose of relaxing such clashes. Furthermore, observed structural changes upon ligand binding are dominated by small motions [51,52], which can be modelled well by energy minimisation [23].

Despite these limitations, substrate-imprinted docking can achieve a high predictive accuracy. As for other docking methods, the choice of the protein structure used for docking is crucial. Lipase structures which are adequate for substrate-imprinted docking must have an accessible substrate binding site and a functional orientation of the side chains in the active site. In the AChE X-ray structure 1VXR and the two CRL X-ray structures 1LPN and 1LPP, the catalytic histidine is considerably displaced by the bound inhibitors. Therefore, for all substrates these structures led to non-productive poses due to a failure of the geometric filter criteria. In contrast to other docking methods, substrate-imprinted docking is robust for other differences in protein structures: X-ray structures of free proteins and inhibitor complexes showed the same predictive accuracy. If the three X-ray structures with a displaced histidine are removed from the dataset, the accuracy of the method is 81%. Thus, substrate-imprinted docking allows to model substrate specificity and in some cases enantioselectivity of lipases and esterases with a good accuracy and with moderate computational and manual effort. The stereoselectivity could be accurately modelled for CALB, where the E-value was very high, while it was not possible to accureately model the stereoselectivity for CRL and BCL, where E-values were lower.

Docking reaction intermediates covalently into enzymes without accounting for flexibility did yield poor results, as can be seen in the results of the conventional docking. Likewise, it has been demonstrated by others, that performing an energy minimisation of ligand-protein complexes without applying filter criteria increased the number of false positives [53]. Thus, all three steps of the substrate-imprinted docking procedure are essential to achieve high accuracy.

### False positive predictions

The conformational changes upon geometry optimisation of the substrate-protein complex often result in a widening of the binding pocket and can lead to false positive docking results in the substrate-imprinted docking approach. It can be argued, that the structures are optimised in a way that would fit any putative substrate used for imprinting whether a substrate or not, resulting in an increase of false positive predictions. This risk of false positives could reduces the ability of substrate-imprinted docking to discriminate between substrates and non-substrates. Previously, it has indeed been shown that energy minimisation of kinase-inhibitor complexes followed by scoring with Autodock resulted in an increase of false positives [53], thus a decreased ability to discriminate between substrates and non-substrates. This shortcoming of flexible protein structures can be counteracted by using more stringent parameters during docking, as we do by using smaller *maximum overlap volumes *in the second round of docking as compared to the first round of docking (1st round: 2.5 Å^3 ^to 7.5 Å^3^, 2nd round: 2.0 Å^3 ^to 3.5 Å^3^), and by applying geometric filter criteria that will discard all non-productive poses, even if they have a good score.

**Figure 2:**
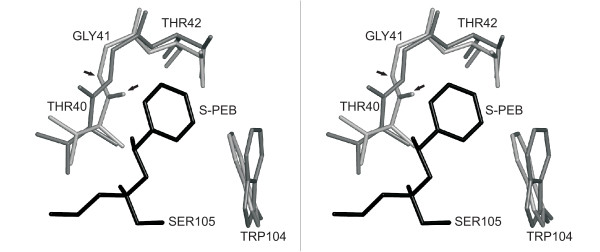
**Binding pocket of C. antarctica lipase B with docked substrate (*S*)-PEB**. Stereo image of the binding pocket of the CALB structure 1LBS (light grey) and the substrate-imprinted model 1LBS/(*S*)-PEB (dark grey) with the covalently bound substrate (*S*)-PEB (black). The backbone of T40 and G41 are twisted (arrows), displacing the backbone oxygen by 2.1 Å.

For CALB and its W104A mutant, the accuracy of docking into the substrate-imprinted structures increased from 60% to 95% when docking into substrate-imprinted structures, and only one false positive result occurred ((*S*)-PEB with 1LBS). This false positive could be identified by analysing the RMSD in the alcohol binding pocket (T40, G41, T42, W104, A281). In this complex, the side chains of W104 and T42 have been displaced by more than 1 Å, and the backbone of T40 and G41 is twisted by almost 90°, thereby displacing the backbone oxygen by 2.1 Å (Fig. 2). This led to a high RMSD in the alcohol pocket (0.68 Å), which considerably exceeded the overall changes in protein structure (0.45 Å). In contrast, for 17 complexes (CALB and its mutant with (*R*)-PEB and (*S*)-PEB) the RMSD of the alcohol binding pocket was in the range of 65% and 121% of their total all-atom RMSD (Table 2). The RMSD of the alcohol pocket exceeded the overall RMSD considerably (170% and 149%) for only one further wild type complex (1LBT/(*S*)-PEB) and one mutant complex (1LBSW104A/(*R*)-PEB), although they were true negatives or positives. Thus, a RMSD exceeding 130% of the overall RMSD can indicate an unreliable optimised structure, which often leads to false predictions. However, this additional analysis also rejects some correct predictions.

Additionally, the increased total accuracy for docking 2- to 4-MPPs into substrate-imprinted CRL and BCL structures was due to a much improved identification of the non-substrates (76%) as compared to docking into the X-ray structures (33%). Therefore we think that the applied docking parameters and filter criteria are suitable to prevent false positives.

### False negative predictions

One major effect of substrate-imprinted docking is the reduction of false negatives. When docking into TcAChE and huBuChE, the number of false negatives is reduced from ten to four by substrate-imprinted docking. In X-ray structures and homology models, the orientation of side chains is not optimised, thus resulting in clashes with docked molecules [54]. Therefore, docking into non-optimised structures resulted in ten false negatives. During geometry optimisation with the covalently bound substrate, the binding pocket adjusted to the substrate. As a result, seven of the ten false negatives did not occur when docking into substrate-imprinted structures. However, one additional false negative occurred when using the substrate-imprinted structures, that did not occur when using the non-optimised structures.

False negative results happen for two reasons. Either no pose for the substrate is found or none of the poses pass the geometric filter criteria. Two false negative results (ACh and BuCh docked into 1P0M) that occurred with both, the substrate-imprinted and the non-optimised structures, are examples for the first case and occurred due to clashes between substrate and protein in the binding pocket. The false negative that occurred with the substrate-imprinted and the conventional docking (ACh docked into 1VXR) is an example for poses that did not pass the geometric filter criteria. In these structures, the binding pocket has adopted a conformation that allows substrate binding, but not in a productive orientation, due to the orientation of the catalytic histidine. In 1VXR, the catalytic histidine has been displaced by the co-crystallised inhibitor [55], which was also the case for the two CRL structures 1LPN and 1LPP. In this conformation catalytic histidine the N_*ϵ *_can not interact with the catalytic serine. With the histidine being unable to form a hydrogen bond to the serine O_*γ*_, the docking pose did not pass the geometric filter criteria and was considered to be non-productive.

The false negative predictions for the huBuChE can be identified by analysing the RMSD of the choline pocket. A comparison of the overall RMSD and the RMSD of the choline pocket after the geometry optimisation revealed that the choline pocket formed by W82, G115, G116, E197, H438, and G439 showed a considerably higher or lower RMSD than the rest of the protein. The all-atom RMSD of the whole protein after geometry optimisation ranged from 0.48 Å to 0.52 Å for huBuChE X-ray structures (Table 6). The RMSD of the choline pocket was 0.29 Å and 0.33 Å for the structure 1P0M, imprinted with ACh and BuCh, which is only 59% and 66% of the total RMSD. The three other substrate-imprinted structures that led to correct docking results had a RMSD for their choline binding pocket between 107% and 113% of the RMSD of the whole structure.

**Table 6:** RMSD of human butyrylcholine esterase after optimization

	All-atom RMSD [Å]	
	total	choline pocket
	
1P0M/ACh	0.49	0.29 (59%)^*a*^
1P0M/BuCh	0.50	0.33 (66%)^*a*^
1XLU/ACh	0.50	0.55 (109%)
1XLU/BuCh	0.48	0.54 (113%)
1XLV/ACh	0.52	0.57 (109%)
1XLV/BuCh	0.50	0.57 (113%)
1XLW/ACh	0.48	0.54 (113%)
1XLW/BuCh	0.52	0.56 (107%)

All-atom RMSD of the choline pocket of huBuChE in comparison to the all-atoms RMSD of the whole protein after geometry optimisation. The percentage values in brackets represent the choline pocket RMSD as a percentage of the all-atoms RMSD of the whole protein. ^*a *^False prediction.

Thus, all false negative predictions of the huBuChE could be identified by a similar method that also identified the false positive docking results for CALB. A RMSD of the relevant binding pocket of the substrate-imprinted structure, that deviates more than 30% from the all-atom RMSD of the whole structure can be used as an indicator for an aberration in the geometry optimisation, resulting in a less reliable docking result.

## Conclusion

Substrate-imprinted enzyme docking combines covalent docking, geometry optimisation, and geometric filter criteria to identify productive substrate poses. For the enzymes examined here, substrate specificity and enantioselectivity of wild type enzymes and mutants were modelled with an accuracy of 81% if the three structures with distorted active site were excluded (68% if the three structures are included). The process consists of five steps:

1. As protein structure, X-ray structures of free enzymes or inhibitor complexes are suitable, as well as reliable homology models. However, it is crucial that the side chains of the catalytic serine and histidine are in a functional orientation.

2. Substrates are covalently docked in a tetrahedral intermediate form at an elevated *maximum overlap volume*. Productive poses are selected by geometric filter criteria and the docking score.

3. The geometry of the selected complexes is optimised by unconstrained energy minimisation.

4. In order to assess the reliability of the optimised structures, the deviation of the structure of the substrate binding site in respect to the overall deviation of the protein during energy minimisation of the complex can be evaluated. Structures where the difference between these deviations is larger than 30%, often led to false positive or false negative predictions.

5. The relaxed protein structure is used for a second round of substrate docking using more stringent docking parameters. Productive poses are again selected by geometric filter criteria and the docking score.

The method seems to be most accurate for modelling substrate specificity and less accurate for modelling enantioselectivity. Substrate-imprinted docking was able to model the differences in substrate specificity of CRL and BCL, and TcAChE and huBuChE, and differences between the enantioselectivity of CALB wild type and its W104A mutant. For CRL and BCL, enantioselectivity could not be reliably modelled.

Substrate-imprinted docking was reproducible and robust toward different X-ray structures of the same protein. Because it combines good accuracy with a moderate computational and manual effort, it is most suited to screen enzyme and mutant libraries with selected substrates.

## Methods

### Preparation of protein structures and substrates

X-ray structures of CALB (1LBS, 1LBT, 1TCA, 1TCB, 1TCC), CRL (1CLE, 1CRL, 1LPM, 1LPN, 1LPO, 1LPP, 1LPS), BCL (2LIP, 3LIP, 4LIP, 5LIP, 1OIL, 1YS1, 1YS2), TcAChE (1CFJ, 1DX6, 1E3Q, 1EVE, 1VXR, 1QIM), and huBuChE (1P0M, 1XLU, 1XLV, 1XLW) were retrieved from the Protein Data Bank [1]. Two CALB structures (1LBS, 1LBT), six CRL structures (1CLE, 1LPM, 1LPN, 1LPO, 1LPP, 1LPS) and four BCL structures (4LIP, 5LIP, 1YS1, 1YS2) had been resolved with a bound inhibitor. From the six selected TcAChE structures, three had been resolved in complex with a large inhibitor (1DX6, 1E3Q, 1EVE), two with a small inhibitor (1CFJ, 1VXR), and one without any inhibitor (1QIM). ¿From the four huBuChE X-ray structures, one had been resolved with a non-covalently bound product molecule (1P0M) and three had been resolved in a covalent complex with a small substrate analogous inhibitor (1XLU, 1XLV, 1XLW). Experimentally, the structures 1VXR, 1LPP, and 1LPN contain inhibitors that caused a very large displacement of the catalytic histidine. These three structures can therefore be considered to be not suited for modelling of catalytic activity, despite having a bound inhibitor, but were included in this study to better assess whether substrate-imprinted docking can correct these structural artefacts or not. Models for the W104A mutant of CALB (1LBSW104A, 1LBTW104A, 1TCAW104A, 1TCBW104A, 1TCCW104A) were created by changing W104 to alanine in the X-ray structures of the wild type using the Swiss-Pdb viewer [56] and selecting the rotamer with the lowest score. W104 is located in the binding pocket for the medium-sized moiety of a secondary alcohol. For the huBuChE structures, the missing residues D378 and D379 were supplemented by MODELLER [57], while keeping all other atoms fixed. The two residues are located on a loop far away from the substrate binding site. These models are referred to as "X-ray structures". The RMSD between two structures was calculated after fitting with the McLachlan algorithm [58], implemented in the program ProFit (A C R Martin, ).

Protonation states of titrateable residues at pH 7 [see Additional file 3, S13] were calculated by TITRA [59], using the Tanford-Kirkwood sphere model, and MEAD [60], using finite-difference methods to solve the Poisson-Boltzmann equation. Both methods predicted the same protonation states for the large majority of all titrateable residues. In few cases (≈2%) where the two methods predicted a different protonation state for the same residue, we relied on the predictions made by TITRA. The catalytic histidine in the protein structure was protonated, because we model a substrate-protein complex where the substrate is covalently bound to the catalytic serine. The formation of the covalent bond between the serine and the substrate ester is the result of a nucleophilic attack of the serine O_*γ *_at the ester carbon. while the proton of the serine is transfered to the histidine.

**Figure 3:**
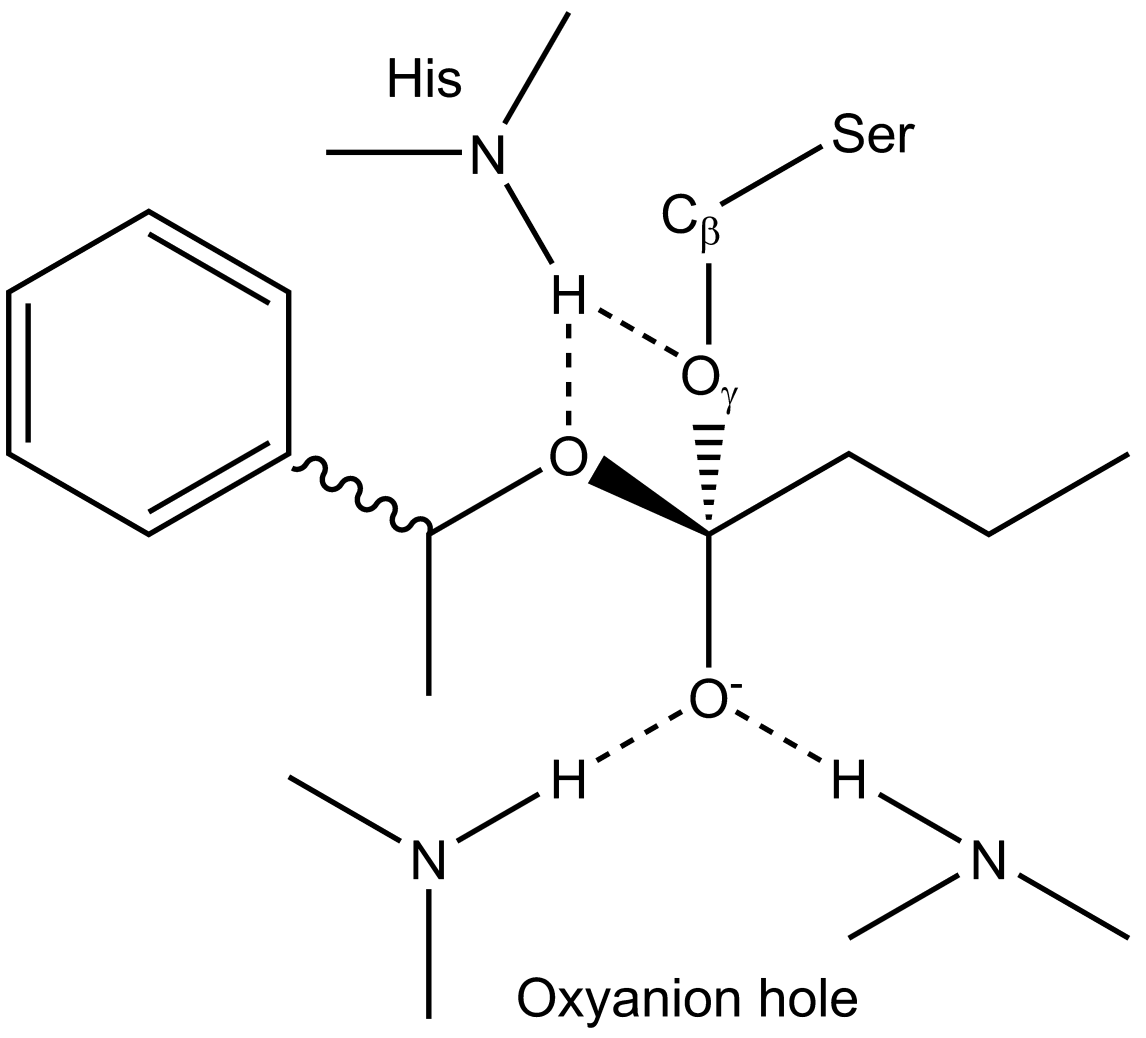
**Substrate in a tetrahedral reaction intermediate form analogous to the transition-state stabilised by the enzyme**. A tetrahedral intermediate form of substrate and enzyme. The activated serine O_*γ *_attacks the carbonly oxygen of the substrate. The transition-state is stabilised by four hydrogen bonds (- - -) between the N-H-groups of the oxyanion hole and the substrate oxyanion, the oxygen of the substrate alcohol moiety and a side chain N-H-groups of the catalytic histidine and between the serine O_*γ *_and a side chain N-H-group of the catalytic histidine. The substrate is docked as a tetrahedral intermediate and includes the O_*γ *_and C_*β *_atoms, which are identical to those of the serine residue.

Substrate esters were constructed as tetrahedral reaction intermediates of the lipase-catalysed ester hydrolysis (Fig. 3), including two atoms of the catalytic serine, which forms a covalent bond to the intermediate. This tetrahedral carbon atom has four substituents – the alkyl moiety, the alcohol moiety, a negatively charged oxygen (oxyanion) and the O_*γ*_-C_*β*_-fragment of the catalytic serine.

### Conventional docking

**Figure 4:**
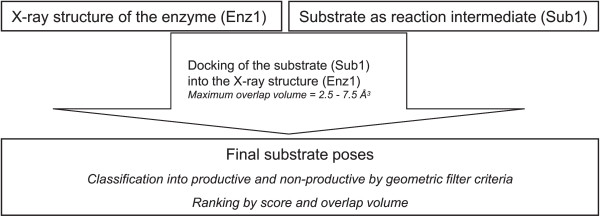
**Flowchart of the conventional docking**. Starting from one enzyme structure (Enz1) and one substrate in a reaction intermediate form (Sub1), the substrate is covalently docked into the structure. The resulting substrate poses are classified according to geometric filter criteria into productive and non-productive, and ranked by docking score.

The conventional docking procedure consists of covalent docking of a reaction intermediate into the X-ray structure of an enzyme with a subsequent scoring and classification of the poses into productive and non-productive ones (Fig. 4). FlexX covalent docking superimposes a fragment (base fragment) of the ligand on a part of the X-ray structure. The base fragment serves as root for the incremental build-up of the whole ligand in the binding pocket. The substrate O_*γ *_and C_*β *_atoms form the base fragment and are superimposed on the O_*γ *_and C_*β *_atoms of the catalytic serine. Up to 50 different conformations of the base fragment are allowed during this superimposition, and the torsion angle of the bond between O_*γ *_and C_*β *_is sampled in a 10° range, according to the default settings of FlexX. The *maximum overlap volume *parameter in FlexX sets a limit for the overlap between the protein and a ligand atom. The allowed average overlap from every ligand atom is 0.4 * *maximum overlap volume*. Poses that exceed any of these values are automatically discarded. During every single conventional docking, the *maximum overlap volume *was gradually increased in 0.5 Å^3 ^steps from 2.5 Å^3 ^to 7.5 Å^3^. Docking with gradually increasing *maximum overlap volumes *is necessary, because the incremental construction algorithm of the ligand used by FlexX [34] can result in some substrate poses that are found at a small *maximum overlap volume*, but not at a larger *maximum overlap volume*, and vice versa. The superimposed atoms of the base fragment and hydrogen atoms are not taken into account in overlap tests, nor is the base fragment considered when clashes between ligand and protein are calculated. The generated substrate poses are classified into productive and non-productive poses by the geometric filter criteria and ranked by score. The geometric filter checks for:(a) the existence of hydrogen bonds between the backbone N-H-groups of the two oxyanion hole residues and the oxyanion of the substrate, (b) a hydrogen bond between a side chain N-H-group of the catalytic histidine and the O_*γ *_of the substrate, and (c) a hydrogen bond between a side chain N-H-group of the catalytic histidine and the oxygen of the alcohol moiety of the substrate (Fig. 3). A substrate pose with those four hydrogen bonds formed is considered to be productive. Hydrogen bonds were identified by FlexX [34,35] and defined according to the pairwise interaction scheme of FlexX. For each group able to act as a hydrogen acceptor or donor, a special interaction surface is defined as part of a sphere centred on the interacting atom. If two interaction centres lie near to each others interaction surface, they form an interaction.

The docking scores given by FlexX are calculated by an empirical scoring function that estimates the free energy of binding [35,61]. The function contains contributions for the loss of entropy, for hydrogen bonds, for ionic and hydrophobic interactions between the protein and the ligand, and for unfavourably close contacts between ligand and protein atoms. A productive pose with a negative score was considered to model a substrate that is converted by the enzyme, while the absence of such a pose was considered to correspond to a non-substrate.

### Substrate-imprinted docking

**Figure 5:**
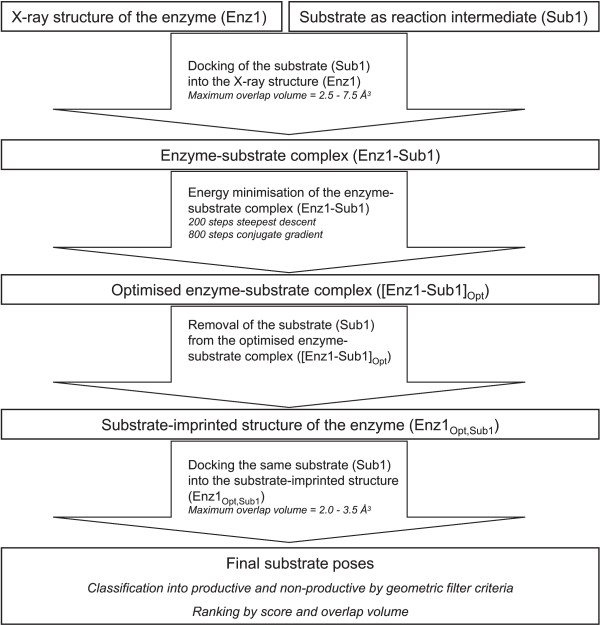
**Flowchart of the substrate-imprinted docking**. Starting from one enzyme structure (Enz1) and one substrate in a reaction intermediate form (Sub1), the substrate is covalently docked into the structure in a first round of docking. The best pose from the first docking is used to construct an enzyme-substrate complex (Enz1-Sub1)), which is then energy minimized and provides an optimised enzyme-substrate complex ([Enz1-Sub1]_*Opt*_). The substrate is removed from this optimised complex, yielding a substrate-imprinted enzyme structure (Enz1_*Opt, Sub1*_). This structure-imprinted structure is used in a second round of docking of the same substrate (Sub1). The resulting substrate poses are classified according to geometric filter criteria into productive and non-productive, and ranked by docking score.

Substrate-imprinted docking consists of a first round of docking by FlexX, a geometry optimisation, a second round of docking, and a final classification and scoring of the resulting poses (Fig. 5). The procedure starts with a X-ray structure and a putative substrate. Stereoisomers of one compound are treated as separate substrates. The putative substrate is covalently docked into the X-ray structure of the enzyme. During this first docking, the *maximum overlap volume *is gradually increased in 0.5 Å^3 ^steps from 2.5 Å^3 ^to 7.5 Å^3^, as described for the conventional docking. A substrate-protein complex is built from the X-ray structure and the pose with the best score by removing the O_*γ *_and C_*β *_atoms of the catalytic serine in the X-ray structure and defining a bond between the C_*β *_atom of the substrate and the C_*α *_atom of the catalytic serine, as described above. If no substrate pose was found during the first round of docking, the method stopped here and the result was considered to be negative. However this occurred only twice in the 236 substrate-imprinted docking runs (2LIP with (*R*)- and (*S*)-MPP). This complex is optimised by energy minimisation (200 steps steepest decent followed by 800 steps conjugate gradient). A new, substrate-imprinted protein structure is extracted from the optimised complex by removing all substrate atoms except for the O_*γ *_and C_*β *_atoms that form the side chain of the catalytic serine. A second round of docking follows, where the same substrate that was used in the first round of docking is covalently docked into the optimised structure. The *maximum overlap volume *parameter is set more stringent in this second docking than in the first docking, and is gradually increased in 0.1 Å^3 ^steps from 2 Å^3 ^to 3.5 Å^3^. All generated substrate poses are scored and classified into productive and non-productive poses as described for the conventional docking. A productive pose with a negative score was considered to model a substrate that is converted by the enzyme, while the absence of such a pose was considered to correspond to a fake substrate, that is not converted by the enzyme.

### Geometry optimisation

In its docked pose, the substrate partially overlaps with the catalytic serine. A substrate-protein complex with the substrate covalently bound to the catalytic serine was created by removing the O_*γ *_and C_*β *_of the catalytic serine and defining a bond between the C_*β *_of the substrate and the C_*α *_of the catalytic serine. Atom types and parameters of the AMBER ff99 force field [62] were used. Parameters and atom types for the new serine-substrate residue were derived by analogy. The partial charges for the serine-substrate residue were assigned with the RESP fit methodology [63] after ab initio geometry optimisation in the gas phase at the Hartree-Fock level of theory with the 6-31G* basis set and calculation of the electrostatic potential in gridpoints according to the Merz-Singh-Kollman scheme [64,65]. Protonation states of titratable residues were used as calculated for the docking steps. Hydrogens were added by LEaP [66]. The system was solvated by placing it in a truncated octahedral water box using the TIP3P water model [67] with a minimal distance of 1 Å between protein and water molecules and a minimal distance of 12 Å between protein and the wall of the box. Counter ions were added in LEaP to neutralise the system. LEaP places the counter ions in a shell around the protein using a Coulombic potential. The protein-ligand complexes were minimised using the AMBER program package [66] and the all-atom AMBER force field ff99. The Sander tool of AMBER was used to perform a 200 step steepest descent minimisation, followed by 800 steps conjugate gradient minimisation in order to relax clashes in the system. Except for the O_*γ *_and C_*β *_atoms that form the serine side chain, all atoms that belong to the substrate were removed from the optimised complex. These structures were referred to as substrate-imprinted structures.

## Abbreviations

ACh: acetylcholine; BCL: *Burkholderia cepacia *lipase; BuCh: butyrylcholine; CALB: *Candida antarctica *lipase B; CRL: *Candida rugosa *lipase; HOB: hydroxyoctanoic acid butyl ester; huBuChE: human butyrylcholine esterase; MDB: methyldecanoic acid butyl ester; MPP: methylpentanoic acid pentyl ester; PEB: 1-phenylethyl butyrate; RMSD: root mean square deviation; TcAChE: *Torpedo californica *acetylcholine esterase.

## Authors' contributions

PT and ST carried out docking and geometry optimisation for TcAChE. PBJ conducted the homology modelling and carried out docking and geometry optimisation for all other enzymes. JP was involved in designing and overseeing the study. All authors have read and approved the final manuscript.

## Supplementary Material

Additional file 1 **docking_scores**. The tables presented here provide the exact docking scores for the conventional and substrate-imprinted docking experiments performed for this study. Click here for file

Additional file 2 **Supplementary Table**. Docking of methylpentanoic acid pentyl esters Click here for file

Additional file 3 **charge_and_protonation**. This file lists the total charge of the proteins used and the protonation states of the residues during docking and energy minimisation. Click here for file
