# Supplementary material for: Modelling substrate specificity and enantioselectivity for lipases and esterases by substrate-imprinted docking
Source: BMC Struct Biol. 2009 Jun 3;9:39. doi: 10.1186/1472-6807-9-39 (PMC2699341; doi:10.1186/1472-6807-9-39)
Supplement: Additional file 1 — docking_scores. The tables presented here provide the exact docking scores for the conventional and substrate-imprinted docking experiments performed for this study. [file 1472-6807-9-39-S1.pdf]

Modelling substrate specificity and enantioselectivity for lipases and esterases by substrate-imprinted docking

Additional file 1 - Docking scores

P Benjamin Juhl, Peter Trodler, Sadhna Tyagi and Jürgen Pleiss

Table S1 - Docking of 2-hydroxyoctanoic acid butyl ester

Docking scores of docking (R)-2-HOB and (S)-2-HOB into seven BCL and seven CRL structures using FlexX. The substrates were docked into the not optimised structures and the substrate-imprinted structures.

	Docking into: non-optimised structures [kJ/mol]		Docking into: substrate-imprinted structures [kJ/mol]	
Structure	(R)-2-HOB	(S)-2-HOB	(R)-2-HOB	(S)-2-HOB
1CLE	-1.4	-1.0	-7.3	-6.8
1CRL	-1.9	-1.8	-9.0	-7.9
1LPM	-3.0	-1.0	-9.0	-7.2
1LPN[b]	n.s.[a]	n.s.	n.s.	n.s.
1LPO	-1.7	-1.8	-5.2	-6.0
1LPP[b]	n.s.	n.s.	n.s.	n.s.
1LPS	n.s.	n.s.	n.s.	-7.1
2LIP	n.s.	n.s.	n.s.	-1.5
3LIP	n.s.	n.s.	-0.8	n.s.
4LIP	-0.2	-0.4	-3.7	-3.3
5LIP	-2.1	-1.6	-2.9	-2.4
1OIL	-0.5	-1.3	-2.1	-2.5
1YS1	-0.4	-2.4	-3.3	-3.5
1YS2	-0.1	-2.1	-3.5	-3.8

[a] no solution, [b] displaced histidine

Table S2 - Docking of 2-methylpentanoic acid pentyl ester

Docking scores of docking (R)-2-MPP and (S)-2-MPP into seven BCL and seven CRL structures using FlexX. The substrates were docked into the not optimised structures and the substrate-imprinted structures.

	Docking into: non-optimised structures [kJ/mol]		Docking into: substrate-imprinted structures [kJ/mol]	
Structure	(R)-2-MPP	(S)-2-MPP	(R)-2-MPP	(S)-2-MPP
1CLE	-4.6	n.s.[a]	n.s.	-9.8
1CRL	-3.0	-1.0	n.s.	n.s.
1LPM	-3.3	-1.7	n.s.	-10.8
1LPN[b]	n.s.	n.s.	n.s.	n.s.
1LPO	-4.9	-1.8	n.s.	n.s.
1LPP[b]	n.s.	n.s.	n.s.	n.s.
1LPS	n.s.	n.s.	n.s.	n.s.
2LIP	n.s.	n.s.	n.s.	n.s.
3LIP	n.s.	n.s.	n.s.	n.s.
4LIP	-0.4	-4.0	-5.4	n.s.
5LIP	-4.0	-1.6	-4.4	n.s.
1OIL	-1.0	-0.9	n.s.	n.s.
1YS1	-1.3	-4.0	-4.6	n.s.
1YS2	-0.8	-3.5	-4.6	n.s.

[a] no solution, [b] displaced histidine

Table S3 - Docking of 3- and 4-methylpentanoic acid pentyl ester

Docking scores of docking (R)-3-MPP, (S)-3-MPP, and 4-MPP into seven BCL and seven CRL structures using FlexX. The substrates were docked into the not optimised structures and the substrate-imprinted structures.

	Docking into: non-optimised structures [kJ/mol]			Docking into: substrate-imprinted structures [kJ/mol]		
Structure	(R)-3-MPP	(S)-3-MPP	4-MPP	(R)-3-MPP	(S)-3-MPP	4-MPP
1CLE	-4.8	-4.7	-6.0	n.s.[a]	n.s.	-12.7
1CRL	-4.5	-6.9	-8.2	n.s.	n.s.	-11.8
1LPM	-3.5	-2.4	-4.4	n.s.	n.s.	-11.8
1LPN[b]	n.s.	n.s.	n.s.	n.s.	n.s.	n.s.
1LPO	-6.0	-6.4	-7.4	n.s.	n.s.	-12.5
1LPP[b]	n.s.	n.s.	n.s.	n.s.	n.s.	n.s.
1LPS	n.s.	n.s.	n.s.	n.s.	n.s.	-9.8
2LIP	n.s.	n.s.	n.s.	n.s.	n.s.	-3.9
3LIP	n.s.	n.s.	-0.0	n.s.	n.s.	-6.8
4LIP	-4.1	-5.4	-4.7	-7.2	-7.3	-5.5
5LIP	-4.6	-3.3	-5.6	n.s.	n.s.	-5.1
1OIL	-1.9	-1.4	-3.5	n.s.	n.s.	-6.2
1YS1	-4.5	-6.1	-5.0	-6.4	-6.7	-5.5
1YS2	-3.9	-4.8	-4.7	-6.4	-6.8	-5.3

[a] no solution, [b] displaced histidine

Table S4 - Docking of 2-methyldecanoic acid butyl ester

Docking scores of docking (R)-2-MDB and (S)-2-MDBP into seven CRL structures using FlexX. The substrates were docked into the not optimised structures and the substrate-imprinted structures.

	Docking into: non-optimised structures [kJ/mol]		Docking into: substrate-imprinted structures [kJ/mol]	
Structure	(R)-2-MDB	(S)-2-MDB	(R)-2-MDB	(S)-2-MDB
1CLE	-2.0	-0.1	-7.8	n.s.[a]
1CRL	n.s.	n.s.	-6.5	-1.0
1LPM	n.s.	n.s.	-6.1	-3.4
1LPN[b]	n.s.	n.s.	n.s.	n.s.
1LPO	-2.9	n.s.	-6.2	-5.6
1LPP[b]	n.s.	n.s.	n.s.	n.s.
1LPS	n.s.	n.s.	n.s.	n.s.

[a] no solution, [b] displaced histidine

Table S5 - Docking of 3-methyldecanoic acid butyl ester

Docking scores of docking (R)-3-MDB and (S)-3-MDBP into seven CRL structures using FlexX. The substrates were docked into the not optimised structures and the substrate-imprinted structures.

	Docking into: non-optimised structures [kJ/mol]		Docking into: substrate-imprinted structures [kJ/mol]	
Structure	(R)-3-MDB	(S)-3-MDB	(R)-3-MDB	(S)-3-MDB
1CLE	-1.5	-1.1	-6.4	n.s.[a]
1CRL	-2.6	-2.9	-7.1	-4.8
1LPM	n.s.	n.s.	-7.7	-7.7
1LPN[b]	n.s.	n.s.	n.s.	n.s.
1LPO	-2.2	-1.2	-7.9	-7.6
1LPP[b]	n.s.	n.s.	n.s.	n.s.
1LPS	n.s.	n.s.	n.s.	n.s.

[a] no solution, [b] displaced histidine

Table S6 - Docking of 4-methyldecanoic acid butyl ester

Docking scores of docking (R)-4-MDB and (S)-4-MDB into seven CRL structures using FlexX. The substrates were docked into the not optimised structures and the substrate-imprinted structures.

	Docking into: non-optimised structures [kJ/mol]		Docking into: substrate-imprinted structures [kJ/mol]	
Structure	(R)-4-MDB	(S)-4-MDB	(R)-4-MDB	(S)-4-MDB
1CLE	-2.5	-2.4	-8.2	-8.8
1CRL	-2.1	-2.1	-10.7	-8.0
1LPM	n.s.[a]	n.s.	-10.7	-8.1
1LPN[b]	n.s.	n.s.	n.s.	n.s.
1LPO	-2.7	-4.3	-8.6	-8.7
1LPP[b]	n.s.	n.s.	n.s.	n.s.
1LPS	n.s.	n.s.	n.s.	-3.4

[a] no solution, [b] displaced histidine

Table S7 - Docking of 5-methyldecanoic acid butyl ester

Docking scores of docking (R)-5-MDB and (S)-5-MDB into seven CRL structures using FlexX. The substrates were docked into the not optimised structures and the substrate-imprinted structures.

	Docking into: non-optimised structures [kJ/mol]		substrate-imprinted structures [kJ/mol]	
Structure	(R)-5-MDB	(S)-5-MDB	(R)-5-MDB	(S)-5-MDB
1CLE	-1.3	-0.7	-8.8	-7.1
1CRL	-1.0	-1.7	-10.8	-11.1
1LPM	n.s.[a]	n.s.	-10.2	-9.9
1LPN[b]	n.s.	n.s.	n.s.	n.s.
1LPO	-2.6	-2.4	-8.7	-7.8
1LPP[b]	n.s.	n.s.	n.s.	n.s.
1LPS	n.s.	n.s.	-8.2	-6.4

[a] no solution, [b] displaced histidine

Table S8 - Docking of 6-methyldecanoic acid butyl ester

Docking scores of docking (R)-6-MDB and (S)-6-MDB into seven CRL structures using FlexX. The substrates were docked into the not optimised structures and the substrate-imprinted structures.

	Docking into: non-optimised structures [kJ/mol]		substrate-imprinted structures [kJ/mol]	
Structure	(R)-6-MDB	(S)-6-MDB	(R)-6-MDB	(S)-6-MDB
1CLE	-2.0	-0.4	-7.6	-7.8
1CRL	-1.7	-0.9	-10.6	-9.7
1LPM	-0.5	-0.2	-9.7	-10.4
1LPN[b]	n.s.[a]	n.s.	n.s.	n.s.
1LPO	-4.2	-1.9	-8.6	-7.9
1LPP[b]	n.s.	n.s.	n.s.	n.s.
1LPS	n.s.	n.s.	-4.7	n.s.

[a] no solution, [b] displaced histidine

Table S9 - Docking of 7-methyldecanoic acid butyl ester

Docking scores of docking (R)-7-MDB and (S)-7-MDB into seven CRL structures using FlexX. The substrates were docked into the not optimised structures and the substrate-imprinted structures.

	Docking into: non-optimised structures [kJ/mol]		Docking into: substrate-imprinted structures [kJ/mol]	
Structure	(R)-7-MDB	(S)-7-MDB	(R)-7-MDB	(S)-7-MDB
1CLE	-0.9	-1.3	-6.1	-7.4
1CRL	-1.2	-1.1	-10.5	-10.3
1LPM	n.s.[a]	n.s.	-9.5	-9.5
1LPN[b]	n.s.	n.s.	n.s.	n.s.
1LPO	-3.1	-2.9	-7.0	-6.7
1LPP[b]	n.s.	n.s.	n.s.	n.s.
1LPS	n.s.	n.s.	n.s.	n.s.

[a] no solution, [b] displaced histidine

Table S10 - Docking of 8-methyldecanoic acid butyl ester

Docking scores of docking (R)-8-MDB and (S)-8-MDB into seven CRL structures using FlexX. The substrates were docked into the not optimised structures and the substrate-imprinted structures.

	Docking into: non-optimised structures [kJ/mol]		Docking into: substrate-imprinted structures [kJ/mol]	
Structure	(R)-8-MDB	(S)-8-MDB	(R)-8-MDB	(S)-8-MDB
1CLE	-1.1	-1.7	-6.3	-8.3
1CRL	-1.1	-0.8	-8.5	-10.2
1LPM	n.s.[a]	n.s.	-8.2	-8.8
1LPN[b]	n.s.	n.s.	n.s.	n.s.
1LPO	-2.8	-2.7	-8.2	-8.2
1LPP[b]	n.s.	n.s.	n.s.	n.s.
1LPS	n.s.	n.s.	n.s.	n.s.

[a] no solution, [b] displaced histidine

Table S11 - Docking of 1-phenylethyl butyrate

Docking scores of docking (R)-PEB and (S)-PEB into five X-ray structures of CALB and five structure models of a W104A mutant of CALB using FlexX. The substrates were docked into the not optimised structures and the substrate-imprinted structures.

	Docking into: non-optimised structures [kJ/mol]		Docking into: substrate-imprinted structures [kJ/mol]	
Structure	(R)-PEB	(S)-PEB	(R)-PEB	(S)-PEB
1LBS	-5.1	n.s.	-11.8	-14.9
1LBSW104A	-4.1	n.s.	-8.4	-9.2
1LBT	-5.9	-5.4	-9.3	n.s.[a]
1LBTW104A	n.s.	n.s.	-8.7	-8.1
1TCA	-1.5	n.s.	-12.3	n.s.
1TCAW104A	-3.9	n.s.	-8.9	-7.4
1TCB	n.s.	n.s.	-8.3	n.s.
1TCBW104A	-4.4	n.s.	-9	-7.6
1TCC	-5	n.s.	-11.4	n.s.
1TCCW104A	-4.5	n.s.	-8.4	-8.1

[a] no solution

Table S12 - Docking of acetylcholine and butyrylcholine

Docking scores of docking ACh BuCh into six X-ray structures of the acetylcholine esterase from *Torpedo californica*, four X-ray structures of the human butyrylcholine esterase and four homology models of the human butyrylcholine esterase using FlexX. The substrates were docked into the not optimised structures and the substrate-imprinted structures.

	Docking into:			
	non-optimised structures [kJ/mol]		substrate-imprinted structures [kJ/mol]	
Structure	ACh	BuCh	ACh	BuCh
1CFJ	-16.0	-13.4	-19.4	n.s.[a]
1DX6	-17.1	-13.8	-20.0	-13.9
1E3Q	n.s.	n.s.	-18.4	n.s.
1EVE	-17.2	-13.3	-20.4	n.s.
1VXR[b]	n.s.	n.s.	n.s.	n.s.
1QIM	-5.5	-2.9	-18.2	n.s.
1P0M	n.s.	n.s.	n.s.	n.s.
1XLU	-13.2	-15.1	-17.7	-15.5
1XLV	-11.6	-13.6	-16.4	-15.1
1XLW	n.s.	n.s.	-14.3	-14.4
1P0Mh	-2.4	-3.6	-14.9	-15.4
1XLUh	-10.5	-10.0	n.s.	-15.2
1XLVh	n.s.	n.s.	-16.0	-14.9
1XLWh	n.s.	n.s.	-15.0	-17.8

[a] no solution, [b] displaced histidine
