# Supplementary material for: Modelling substrate specificity and enantioselectivity for lipases and esterases by substrate-imprinted docking
Source: BMC Struct Biol. 2009 Jun 3;9:39. doi: 10.1186/1472-6807-9-39 (PMC2699341; doi:10.1186/1472-6807-9-39)
Supplement: Additional file 2 — Supplementary Table. Docking of methylpentanoic acid pentyl esters [file 1472-6807-9-39-S2.pdf]

Docking of methylpentanoic acid pentyl esters

	Docking into:									
	X-ray structures					Substrate-imprinted structures				
Structure	(R)-2-MPP	S)-2-MPP	(R)-3-MPP	(S)-3-MPP	4-MPP	(R)-2-MPP	(S)-2-MPP	(R)-3-MPP	(S)-3-MPP	4-MPP
Candida rugosa lipase										
Experimental data	+	+	-	-	+	+	+	-	-	+
1CLE[a]	**+**	-	+	+	**+**	-	**+**	**-**	**-**	**+**
1CRL	**+**	**+**	+	+	**+**	-	-	**-**	**-**	**+**
1LPM[a]	**+**	**+**	+	+	**+**	-	**+**	**-**	**-**	**+**
1LPN[a,b]	-	-	**-**	**-**	-	-	-	**-**	**-**	-
1LPO[a]	**+**	**+**	+	+	**+**	-	-	**-**	**-**	**+**
1LPP[a,b]	-	-	**-**	**-**	-	-	-	**-**	**-**	-
1LPS[a]	-	-	**-**	**-**	-	-	-	**-**	**-**	**+**
No. false predictions	3	4	4	4	3	7	5	0	0	2
Burkholderia cepacia lipase										
Experimental data	-	-	-	-	+	-	-	-	-	+
2LIP	**-**	**-**	**-**	**-**	-	**-**[c]	**-**[c]	**-**	**-**	**+**
3LIP	**-**	**-**	**-**	**-**	**+**	**-**	**-**	**-**	**-**	**+**
4LIP[a]	+	+	+	+	**+**	+	**-**	+	+	**+**
5LIP[a]	+	+	+	+	**+**	+	**-**	**-**	**-**	**+**
1OIL	+	+	+	+	**+**	**-**	**-**	**-**	**-**	**+**
1YS1[a]	+	+	+	+	**+**	+	**-**	+	+	**+**
1YS2[a]	+	+	+	+	**+**	+	**-**	+	+	**+**
No. false predictions	5	5	5	5	1	4	0	3	3	0

Docking of (R)-2-MPP, (S)-2-MPP, (R)-3-MPP, (S)-3-MPP, and 4-MPP into seven BCL and seven CRL structures using FlexX. The substrates were docked into the not optimised X-ray structures and the substrate-imprinted structures. "+" and "-" indicate that the docking results predict MPP to be a substrate or a non-substrate. Correct predictions are indicated by bold and large font type. Experimental data [43] is included for comparison. [a] Structure was resolved with an inhibitor bound. [b] Displaced histidine. [c] No substrate pose was found during the first round of docking to construct the substrate-protein complex.
