# Supplementary material for: Modelling substrate specificity and enantioselectivity for lipases and esterases by substrate-imprinted docking
Source: BMC Struct Biol. 2009 Jun 3;9:39. doi: 10.1186/1472-6807-9-39 (PMC2699341; doi:10.1186/1472-6807-9-39)
Supplement: Additional file 3 — charge_and_protonation. This file lists the total charge of the proteins used and the protonation states of the residues during docking and energy minimisation. [file 1472-6807-9-39-S3.pdf]

Modelling substrate specificity and enantioselectivity for lipases and esterases by substrate-imprinted docking

Additional file 3 - Charge and protonation of the proteins

P Benjamin Juhl, Peter Trodler, Sadhna Tyagi and Jürgen Pleiss

Table S13 - Charge and protonation of the proteins

Residues were protonated for a pH of 7 according to their calculated pKa values. Most titrateable residues had the same protonation state the would have had with their intrinsic pKa value and are therefore not listed. The catalytic histidine was always protonated as explained in the methods section.

Protein	Total charge	Protonated residues
CALB	0	His224
CRL	-16	His368, His449
BCL	-2	His86, His286
AChE	-5	His422, His437, His468
BuChE	+2	His423, His438
